# Measurement and Structure of Cognition in the Longitudinal Aging Study in India—Diagnostic Assessment of Dementia

**DOI:** 10.1093/geroni/igaa057.2280

**Published:** 2020-12-16

**Authors:** Alden Gross, Pranali Khobragade, Erik Meijer, Judith Saxton

**Affiliations:** 1 Johns Hopkins Bloomberg School of Public Health, Baltimore, Maryland, United States; 2 University of southern california, Los Angeles, California, United States

## Abstract

We tested whether a complex model of human cognitive abilities based on Cattell-Horn-Carroll (CHC) theory, developed in English-speaking samples, adequately describes correlations among tests in the Longitudinal Aging Study in India-Diagnostic Assessment of Dementia (LASI-DAD) (N=3,224). Tests in the neuropsychological battery were chosen for their appropriateness for measuring cognition in older adults in India and suitability for co-calibration with the core LASI survey (N=72,000). We evaluated the factor structure and its conformity with a classical CHC factor model incorporating measurement models for general cognition, 5 broad domains (orientation, executive functioning, language/fluency, memory, visuospatial), and 5 narrow domains (abstract reasoning, attention/speed, immediate memory, delayed memory, recognition memory) of cognitive performance. Model fit was adequate (RMSEA:0.051; CFI:0.916; SRMR:0.060). We demonstrated configural factorial invariance of a cognitive battery in the Indian LASI-DAD using CHC theory. Broad domain factors may be used to rank individuals with respect to cognitive performance and classify cognitive impairment.

